# A case of duodenal duplication cyst mimicking a pancreatic pseudocyst with intracystic hemorrhage

**DOI:** 10.1186/s40792-019-0644-3

**Published:** 2019-05-27

**Authors:** Sotaro Fukuhara, Saburo Fukuda, Hiroyuki Sawada, Masayuki Shishida, Sho Ishikawa, Kohata Akihiro, Azusa Kai, Yuzoh Hirata, Seiji Fujisaki, Mamoru Takahashi, Hideto Sakimoto

**Affiliations:** 1 Department of Surgery, Chugoku Rousai Hospital, 1-5-1 Hirotagaya, Kure, Hiroshima, 737-0193 Japan; 2 Department of Gastroenterological and Transplant Surgery, Applied Life Sciences, Institute of Biomedical and Health Sciences, Hiroshima University, 1-2-3 Kasumi, Hiroshima, 734-8551 Japan

**Keywords:** Duodenum duplication cyst, Pancreatic pseudocyst, Intracystic hemorrhage

## Abstract

**Background:**

Duodenal duplication cysts in adults are rare, and a preoperative diagnosis remains difficult because clinical manifestations are nonspecific and variable. We describe a case of a duodenal duplication cyst mimicking a pancreatic pseudocyst with repeated intracystic hemorrhage.

**Case presentation:**

A 47-year-old male who complained of upper abdominal pain and vomiting was referred to our hospital. He was a heavy drinker and had a past history of hospitalization for alcoholic chronic pancreatitis. Plain abdominal computed tomography (CT) showed a cystic lesion of 7 cm in size in the lumen near the second part of the duodenum. The cystic lesion showed high density inside. Gastrointestinal endoscopy revealed that the lumen of the duodenum was deformed by a submucosal tumor-like mass and the endoscope could not pass through it, but active bleeding was not seen in the lumen of the duodenum. On the fourth day of hospitalization, anemia progressed and contrast-enhanced CT demonstrated a high-density spot on the wall of the cystic lesion. A pancreatic pseudocyst complicated with intracystic hemorrhage was preliminarily considered. Angiography was immediately performed, and a pseudoaneurysm was identified in the branch of the anterior superior pancreaticoduodenal artery (ASPDA). Transcatheter arterial embolization (TAE) was performed. Anemia did not progress after that, and follow-up CT showed reduction in the size of the cystic lesion. Afterward, the same symptoms recurred twice and surgical treatment was performed for the pancreatic pseudocyst with repeated intracystic hemorrhage. Macroscopically, a cystic mass of 5 cm in size was adjacent to the second part of the duodenum on the pancreas side. A pinhole-sized communication was identified between the cyst and the duodenum lumen. Microscopically, the cyst wall was composed of gastric mucosa and shared a common proper muscle layer with the duodenum. Chronic ulcers and erosions were seen in the cyst. Based on these findings, a diagnosis of duodenal duplication cyst was made.

**Conclusions:**

Duodenal duplication cysts in adults have seldom been reported and should be considered as a differential diagnosis for a patient with a cystic lesion adjoining the duodenum.

## Background

Duplication cysts are rare congenital anomalies of the gastrointestinal tract. Duodenal duplication cysts are rare, accounting for only 5 to 7% of gastrointestinal tract duplication cysts [1]. They are usually diagnosed in infancy or early childhood. Duodenal duplication cysts in adults are rare and remain difficult to diagnose. Pancreatic pseudocyst formation is a well-known complication in chronic and acute pancreatitis. On rare occasions, life-threatening complications such as intracystic hemorrhage occur. Herein, we describe a rare case of a duodenal duplication cyst in an adult mimicking a pancreatic pseudocyst with repeated intracystic hemorrhage.

## Case presentation

**Fig. 1:**
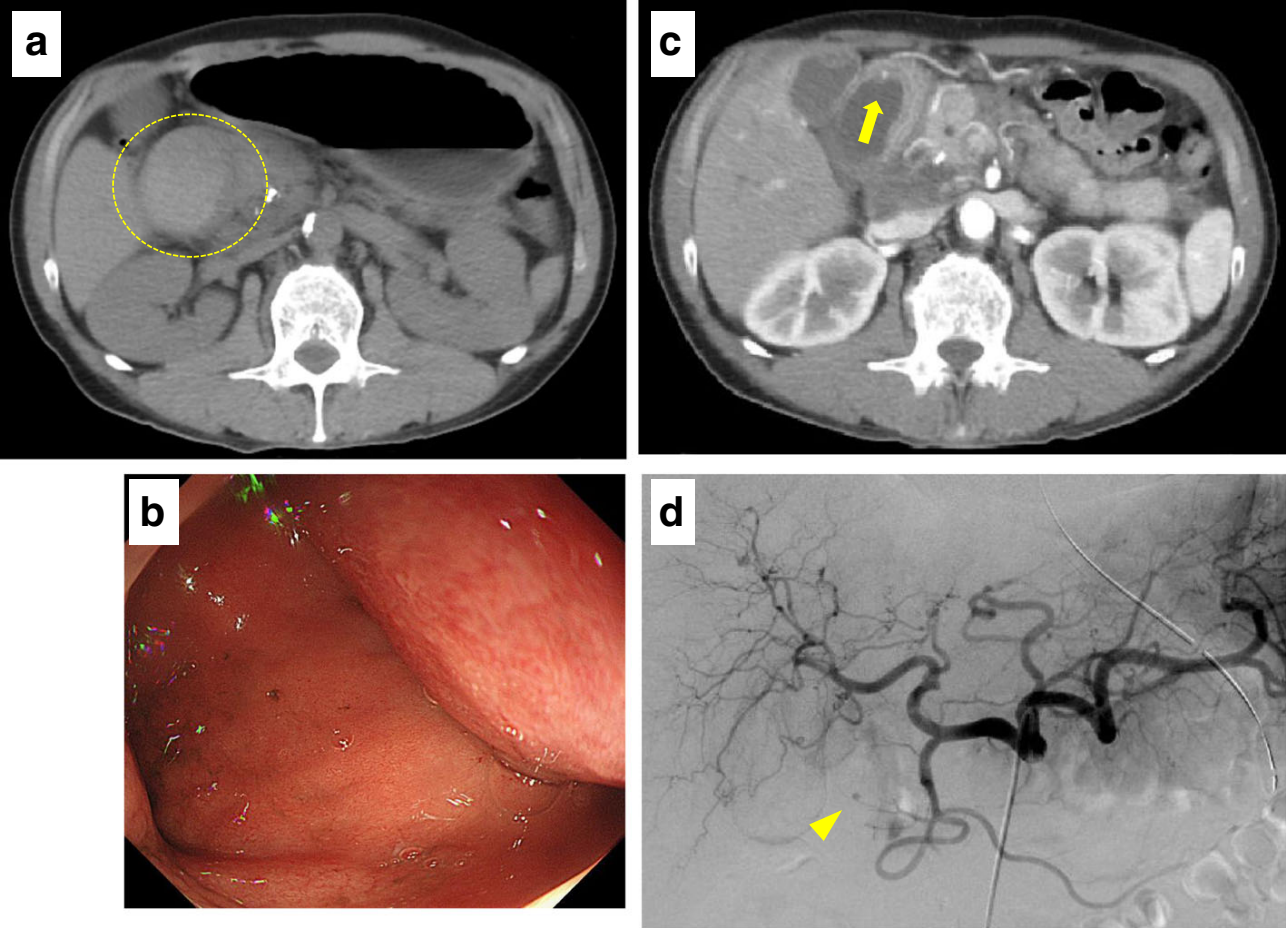
**a** Plain abdominal computed tomography (CT) showed a cystic lesion of 7 cm in size in the lumen near the second part of duodenum. The cystic lesion showed high density inside. **b** Gastrointestinal endoscopy revealed that the lumen of the duodenum was deformed by a submucosal tumor-like mass. Active bleeding was not seen in the lumen of the duodenum. **c** Contrast-enhanced CT demonstrated a high-density spot on the wall of the cystic lesion (yellow arrow). **d** A pseudoaneurysm was identified in the branch of the anterior superior pancreaticoduodenal artery (ASPDA) by angiography (yellow arrowhead)

**Fig. 2:**
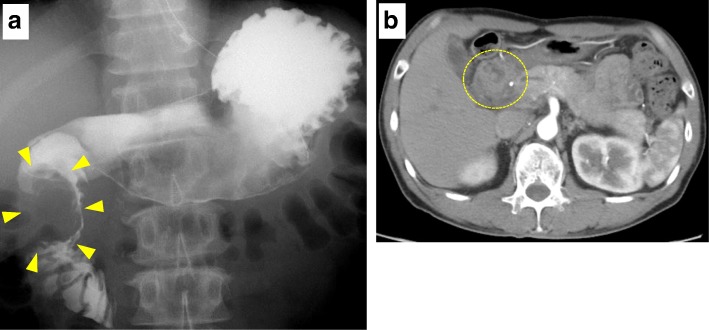
**a** Upper gastrointestinal series demonstrated a filling defect in the duodenum, while the inside of the cystic lesion was not contrasted. **b** Follow-up CT on the 27th day after TAE showed that the cyst decreased in size

A 47-year-old male who complained of upper abdominal pain and vomiting was referred to our hospital. He was a heavy drinker and had a past history of hospitalization for alcoholic chronic pancreatitis. Laboratory data revealed elevated levels of amylase (245 IU/L), CRP (14.99 mg/dl), and white blood cell count (14900/μL). Plain abdominal computed tomography (CT) showed a cystic lesion of 7 cm in size in the lumen near the second part of the duodenum. The cystic lesion showed high density inside. The pancreas was slightly enlarged, and the main pancreatic duct was dilated. Calcifications were seen in the uncus of the pancreas (Fig. 1a). Gastrointestinal endoscopy revealed that the lumen of the duodenum was deformed by a submucosal tumor-like mass and the endoscope could not pass through it (Fig. 1b). However, active bleeding was not seen in the lumen of the duodenum. A submucosal tumor or hematoma of the duodenum or a pancreatic pseudocyst associated with chronic pancreatitis was suspected. On the fourth day of hospitalization, his hemoglobin level had decreased from 14.0 to 11.1 g/dl. Contrast-enhanced CT demonstrated a high-density spot on the wall of the cystic lesion (Fig. 1c). A pancreatic pseudocyst complicated with intracystic hemorrhage was preliminary considered. Angiography was immediately performed, and a pseudoaneurysm was identified in the branch of the anterior superior pancreaticoduodenal artery (ASPDA) (Fig. 1d). The pseudoaneurysm was successfully treated with transcatheter arterial embolization (TAE). Anemia did not progress after that. Upper gastrointestinal series demonstrated a filling defect in the duodenum, while the inside of the cystic lesion was not contrasted (Fig. 2a). Magnetic resonance cholangiopancreatography (MRCP) was performed but did not show a communication between the cyst and the pancreatic and biliary ducts. Follow-up CT on the 27th day after TAE showed that the cyst had decreased in size to 2 cm and obstruction of the duodenum was gradually improved (Fig. 2b). Surgical treatment was considered for the pancreatic pseudocyst with intracystic hemorrhage. However, he refused an operation and was discharged on the 34th day after TAE.

**Fig. 3:**
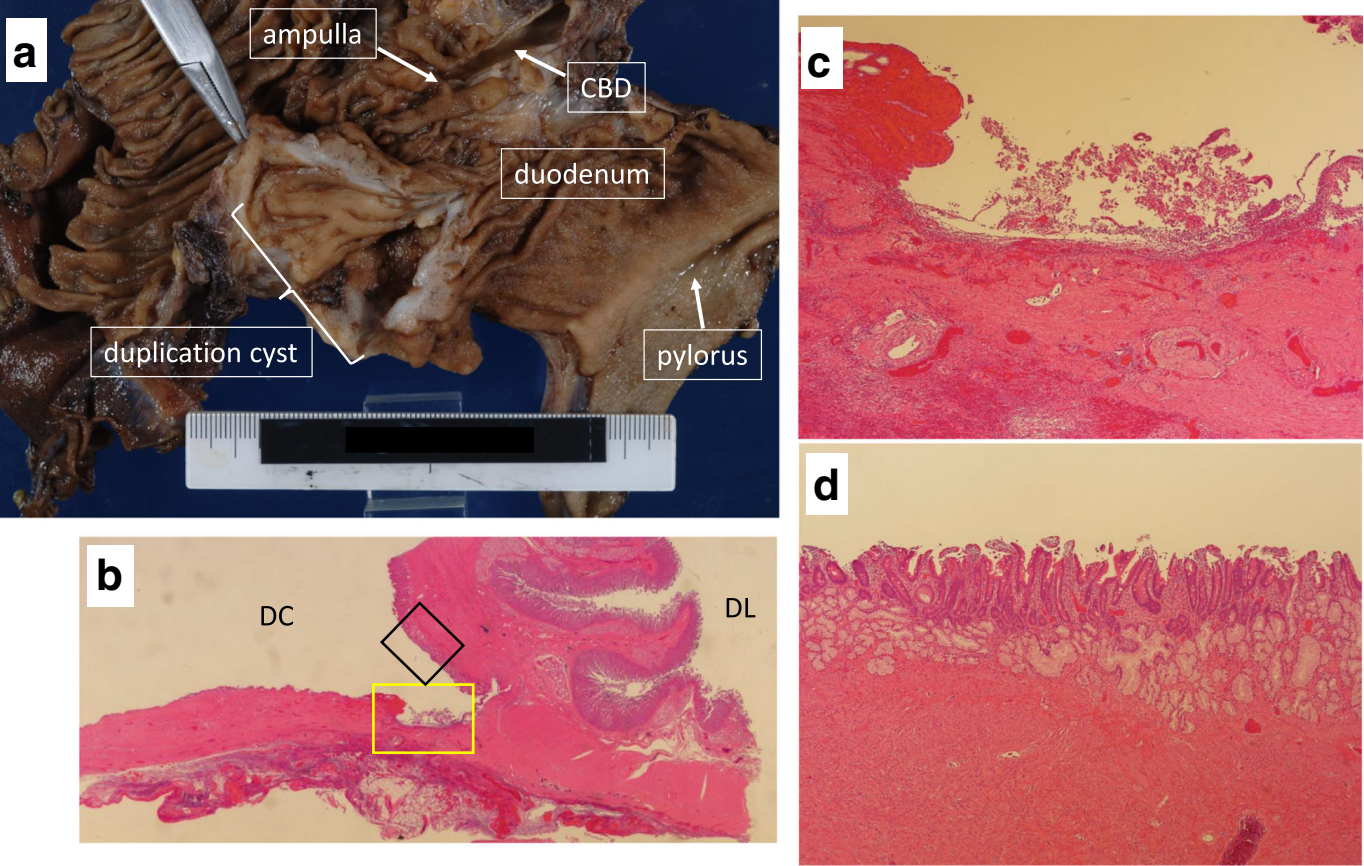
**a** Macroscopically, a cystic mass of 5 cm in size was adjacent to the second part of the duodenum on the pancreas side and was close to the ampulla. **b** Microscopically, the wall of the cyst was composed of gastric mucosa and shared a common proper muscle layer with the duodenum. DL indicates the side of the duodenal lumen, and DC indicates the side of duplication cyst (H&E × 1.1). **c** Magnified view of the yellow frame area in **b**. Chronic ulcers and erosions were seen in the cyst (H&E × 40). **d** Magnified view of the black frame area in **b**. Ectopic gastric mucosa was observed in non-ulcerative region (H&E × 100)

Two years later, abdominal pain and vomiting recurred. The cyst was enlarged again, and CT showed that it contained high-density fluid. Recurrence of a pancreatic pseudocyst with intracystic hemorrhage was suspected because of anemia progression. TAE was performed in the branches of the ASPDA and posterior superior pancreaticoduodenal artery (PSPDA). After TAE, the size of the cyst decreased and symptoms were relieved. However, the same symptoms recurred 2 months later. We obtained informed consent for surgical treatment, and we performed subtotal stomach-preserving pancreatoduodenectomy (SSPPD). Intraoperatively, severe inflammatory adhesion was noted around the pancreas head and the border between the pancreas and the cystic lesion was unclear. Macroscopically, a cystic mass of 5 cm in size was adjacent to the second part of the duodenum on the pancreas side and was close to the ampulla (Fig. 3a). A pinhole-sized communication was identified between the cyst and the duodenum lumen. Microscopically, the cyst was filled with mucus and the wall of the cyst was composed of gastric mucosa and shared a common proper muscle layer with the duodenum. Chronic ulcers and erosions were seen in the cyst (Fig. 3b, c). Ectopic gastric mucosa was observed in non-ulcerative region (Fig. 3d). Based on these findings, a diagnosis of duodenal duplication cyst was made. The patient’s postoperative course was uneventful, and he was discharged on the 30th day after the operation.

## Discussion

Duplication cysts are rare congenital anomalies that can occur anywhere along the alimentary tract, and they are defined by the presence of epithelial mucosal lining, a well-developed smooth muscle layer, and an intimate attachment to the native gastrointestinal tract [2]. Duplication cysts are located most often in the distal ileum, followed by the esophagus, colon, and jejunum. Duodenal duplication cysts account for only 5 to 7% of all gastrointestinal tract duplication cysts [3]. Chen et al. reviewed 47 cases of duodenal duplication cysts [1]. Males and females were equally affected. The majority of cases were diagnosed in infancy or early childhood. In rare cases, duodenal duplication cysts can remain asymptomatic until adulthood, and 38% of patients are diagnosed after the age of 20 years. The clinical manifestations are nonspecific and variable. The most common manifestations are abdominal pain, nausea, and vomiting. Other manifestations such as gastrointestinal obstruction, intussusception, infection, pancreatitis, and gastrointestinal bleeding have also been reported [4–7]. Duodenal duplication cysts are mostly located in the second and third portion of the duodenum, and they may communicate with the native duodenum lumen or the pancreatic or biliary duct [1]. Clinical manifestations are related to the size and location of the lesion and whether the lesion is communicating or non-communicating. Another distinctive feature is that 25 to 35% of duplication cysts contain ectopic mucosa [8]. The presence of ectopic gastric mucosa can cause the development of complications such as bleeding and peptide ulcer.

Preoperative diagnosis of a duodenal duplication cyst is difficult. CT is useful for determining the size and location of a cystic lesion. MRCP and endoscopic retrograde cholangiopancreatography (ERCP) are useful for visualizing the pancreaticobiliary tract and its relationship to the cystic lesion. However, all of the modalities only enable suspicion of the presence of an abnormal lesion, and the diagnosis is confirmed by laparotomy and pathologic results. Recently, endoscopic ultrasonography (EUS) has been reported as safe and accurate for diagnosis of duplication cysts. The typical appearance on sonographic examination is a “muscular rim sign” manifesting as inner echogenic mucosa and outer hypoechoic muscle layer. Muscular peristalsis of the cyst wall noted on EUS helps distinguish duplication cyst from other abdominal cysts [1, 9]. Unfortunately, EUS was not performed in our case.

Differential diagnosis can include any cystic masses in the pancreaticoduodenal region. Among them, special attention is needed for choledochoceles, intraluminal duodenal diverticulum, and pancreatic pseudocyst [10]. A choledochocele, especially type III by Todani, is an isolated cystic dilatation of the distal portion of the common bile duct. Histologically, choledochoceles are lined by either bile duct or gallbladder mucosa [11]. Intraluminal duodenal diverticulum is considered to be an incomplete mucosal septum. It can be discriminated from a duodenal duplication cyst by the lack of submucosal and muscular layers [12]. It has been reported that a barium-filled “windsock” lying in the second portion of the duodenum is the characteristic radiographic appearance of upper gastrointestinal series [13]. A pancreatic pseudocyst is a complication that can arise in both acute pancreatitis and chronic pancreatitis. When a duplication cyst is located within the pancreas, the cystic lesion is frequently interpreted as a pseudocyst. Intracystic hemorrhage associated with pancreatitis is one of the life-threatening complications. The reported mortality rate ranges from 15 to 50% [14]. Optimal therapeutic strategy of pancreatic pseudocyst with intracystic hemorrhage remains controversial, but TAE is considered as the first-line therapy to stop the bleeding. When TAE fails or rebleeding develops after TAE, surgical treatment should be considered for pancreatic pseudocyst complicated with intracystic hemorrhage [15].

In our case, the duplication cyst had a pinhole-sized communication with the native duodenum lumen, and the pinhole size was too small to be observed by endoscopy. Upper gastrointestinal series demonstrated a filling defect in the duodenum, but the inside of the cystic lesion was not contrasted. We regarded this radiographic finding as severe compression by the pancreatic pseudocyst outside of the duodenum. In addition, the patient had a past history of alcoholic chronic pancreatitis. Based on these findings, we had no doubts that the cystic mass was a pancreatic pseudocyst associated with chronic pancreatitis before surgery. Consequently, a high-density spot within the cyst that was demonstrated by contrast-enhanced CT was thought to be intracystic hemorrhage. At first, TAE was performed for hemostasis. After all, surgical resection was performed because intracystic hemorrhage repeated after TAE. Pathological examination revealed that the cystic lesion was a duodenal duplication cyst and that the hemorrhage resulted from peptide ulceration of the ectopic gastric mucosa within the cyst. The mechanism why the cyst was decreased in size after TAE is thought that hemorrhage within the cyst was stopped by TAE and the accumulated blood in the cyst was drained from a pinhole-sized communication into duodenum lumen and the cyst resulted in shrinkage.

Optimal treatment for a duodenal duplication cyst is complete resection, thus preventing any further risk that may arise from residual ectopic gastric mucosa as well as the possibility of the duplication cyst becoming malignant [16]. Surgical management is decided by the size, location, and proximity to the duodenal wall or pancreaticobiliary duct. When duodenal cysts are small and are not associated with the pancreatic and bile ducts or the head of the pancreas, total excision is the procedure of choice. When the duodenal cysts are located in close proximity to the ampulla, pancreaticoduodenectomy (PD) is sometimes necessary [1, 5]. PD should remain an ultimate option because of high morbidity and mortality. Alternatively, partial resection, marsupialization, mucosal stripping, or internal derivation has been recommended [16]. In our case, PD might have been over-surgery for the patient with benign disease of duodenal duplication. The reasons why we performed PD are as follows: First, the duodenal duplication cyst was initially misdiagnosed as a pancreatic pseudocyst. Second, as the resected specimen shows, the duplication cyst was close to the ampulla and severe inflammatory adhesion around the pancreas head was noted during the operation. Even if we had made an accurate preoperative diagnosis with a duplication cyst, it seems to be rather difficult to perform the operation like partial resection of the duodenum. Third, this case was a symptomatic duodenal duplication cyst complicated with repeated hemorrhage. Therefore, complete resection of the cyst was needed. From these reasons, it seems that PD was feasible as a surgical procedure for this case.

## Conclusions

We described a rare case of a duodenal duplication cyst mimicking a pancreatic pseudocyst with intracystic hemorrhage. Duodenal duplication cysts in adults have seldom been reported and should be considered as a differential diagnosis for patients with a cystic lesion adjoining the duodenum.

## Data Availability

The authors declare that all data in this article are available within the article

## Abbreviations

ASPDA: Anterior superior pancreaticoduodenal artery
CRP: C-reactive protein
CT: Computed tomography
ERCP: Endoscopic retrograde cholangiopancreatography
EUS: Endoscopic ultrasonography
MRCP: Magnetic resonance cholangiopancreatography
MRI: Magnetic resonance imaging
PD: Pancreatoduodenectomy
PSPDA: Posterior superior pancreaticoduodenal artery
SSPPD: Subtotal stomach-preserving pancreatoduodenectomy
TAE: Transcatheter arterial embolization
